# The MIOREX complex - lean management of mitochondrial gene expression

**DOI:** 10.18632/oncotarget.4783

**Published:** 2015-07-10

**Authors:** Kirsten Kehrein, Braulio Vargas Möller-Hergt, Martin Ott

**Affiliations:** Department of Biochemistry and Biophysics, Center for Biomembrane Research, Stockholm University, Stockholm, Sweden

The inner membrane of mitochondria accommodates the oxidative phosphorylation complexes that are composed of subunits encoded and synthesized in two different cellular compartments, with most of the subunits expressed from nuclear DNA. Interestingly, mitochondrial gene expression provides only a handful of proteins that nevertheless represent the catalytic core subunits of the oxidative phosphorylation machinery, rendering mitochondrial translation a crucial process for cellular metabolism. Because mitochondria developed from a prokaryotic ancestor it has long been assumed that mitochondrial gene expression might employ similar mechanisms as found in bacteria. However, recent work has revealed many novel organelle-specific aspects that probably evolved to facilitate and optimize gene expression in the organelle.

Mitochondria contain a full genetic system with DNA, mitochondrial ribosomes and all factors required to: A) replicate and transcribe the DNA, B) mature and turnover mRNAs, tRNAs and catalytic RNA, and C) assemble mitochondrial ribosomes. Moreover, mitochondria-specific ribosomes are responsible for the synthesis of the hydrophobic core subunits of the oxidative phosphorylation system. How all these factors are organized in time and space and coordinated with nuclear gene expression is a busy area of mitochondrial research. Indeed, recent work shows that the mitochondrial gene expression machinery is much higher organized than previously assumed. Studies in the mammalian system showed that factors involved in RNA metabolism and ribosome assembly are present in distinct foci, termed RNA granules, that are found in close proximity to the mtDNA [1–3, 5]. These assemblies are thought to connect transcription and maturation of the RNA with downstream processes like ribosome assembly, tRNA modifications and mRNA maturation.

The organization of mitochondrial gene expression might extend far beyond these RNA granules. By developing a system allowing the purification of intact mitochondrial ribosomes, we could demonstrate that mitochondrial ribosomes in yeast form large expressosome-like assemblies that gather almost all factors involved in posttranscriptional steps of gene expression that we termed MIOREX complexes [4]. Some of those complexes further co-localize with the mitochondrial nucleoid thereby consolidating all steps of gene expression (nucleoid-MIOREX). This higher organization of mitochondrial ribosomes might has evolved to bring all necessary factors in close proximity, making the whole process of gene expression faster and more efficient. Hence, the close coupling of transcription, mRNA maturation and translation with oxidative phosphorylation complex assembly allows the channeled transport of mRNAs like in a construction-line. This RNA channeling on the ribosome represents a completely novel form of translation organization. However, it is still an intriguing question how the two types of MIOREX complexes are functionally connected and what specific role either of the complexes has.

One hypothesis is that the peripheral-MIOREX complexes might help to coordinate translation with oxidative phosphorylation complex assembly in a system where gene expression is not regulated at the level of transcription. We and others have recently identified feedback loops operating in the mitochondrial matrix that connect translation of key respiratory chain subunits with the efficiency of their assembly [6, 7]. In cases where assembly is blocked, translation of these mRNAs is reduced, and stimulated in case the mitochondrially encoded subunits are properly assembled. This elegant system directly monitors the efficiency of assembly to coordinate mitochondrial gene expression with the influx of nuclear encoded subunits allowing the cell to optimally invest resources.

The careful organization and channeled transfer of mRNAs in the MIOREX complexes reflects a common theme in mitochondrial biology: Many processes are organized in large assemblies making subsequent biochemical reactions more efficient. Surely, it will be a long, challenging and exciting way to unravel the unique features that mitochondria evolved to express a handful of genes and to learn which other features mitochondria acquired in their journey from a prokaryotic endosymbiont to a “modern” organelle. Because most mitochondrial diseases arise from aberrant mitochondrial gene expression, insights into this fascinating system will allow to develop new therapeutic strategies for these often fatal diseases.
